# Dynamics of HIV DNA reservoir seeding in a cohort of superinfected Kenyan women

**DOI:** 10.1371/journal.ppat.1008286

**Published:** 2020-02-05

**Authors:** Mark D. Pankau, Daniel B. Reeves, Elias Harkins, Keshet Ronen, Walter Jaoko, Kishor Mandaliya, Susan M. Graham, R. Scott McClelland, Frederick A. Matsen IV, Joshua T. Schiffer, Julie Overbaugh, Dara A. Lehman

**Affiliations:** 1 Human Biology Division, Fred Hutchinson Cancer Research Center, Seattle, WA, United States of America; 2 Department of Global Health, University of Washington, Seattle, WA, United States of America; 3 Vaccine and Infectious Disease Division, Fred Hutchinson Cancer Research Center, Seattle, WA, United States of America; 4 Division of Public Health Sciences, Fred Hutchinson Cancer Research Center, Seattle, WA, United States of America; 5 Department of Medical Microbiology, University of Nairobi, Kenyatta National Hospital, Nairobi, Kenya; 6 Coast Provincial General Hospital, Women’s Health Project, Mombasa, Kenya; 7 Department of Medicine, University of Washington, Seattle, WA, United States of America; 8 Department of Epidemiology, University of Washington, Seattle, WA, United States of America; Emory University, UNITED STATES

## Abstract

A reservoir of HIV-infected cells that persists despite suppressive antiretroviral therapy (ART) is the source of viral rebound upon ART cessation and the major barrier to a cure. Understanding reservoir seeding dynamics will help identify the best timing for HIV cure strategies. Here we characterize reservoir seeding using longitudinal samples from before and after ART initiation in individuals who sequentially became infected with genetically distinct HIV variants (superinfected). We previously identified cases of superinfection in a cohort of Kenyan women, and the dates of both initial infection and superinfection were determined. Six women, superinfected 0.2–5.2 years after initial infection, were subsequently treated with ART 5.4–18.0 years after initial infection. We performed next-generation sequencing of HIV *gag* and *env* RNA from plasma collected during acute infection as well as every ~2 years thereafter until ART initiation, and of HIV DNA from PBMCs collected 0.9–4.8 years after viral suppression on ART. We assessed phylogenetic relationships between HIV DNA reservoir sequences and longitudinal plasma RNA sequences prior to ART, to determine proportions of initial and superinfecting variants in the reservoir. The proportions of initial and superinfection lineage variants present in the HIV DNA reservoir were most similar to the proportions present in HIV RNA immediately prior to ART initiation. Phylogenetic analysis confirmed that the majority of HIV DNA reservoir sequences had the smallest pairwise distance to RNA sequences from timepoints closest to ART initiation. Our data suggest that while reservoir cells are created throughout pre-ART infection, the majority of HIV-infected cells that persist during ART entered the reservoir near the time of ART initiation. We estimate the half-life of pre-ART DNA reservoir sequences to be ~25 months, which is shorter than estimated reservoir decay rates during suppressive ART, implying continual decay and reseeding of the reservoir up to the point of ART initiation.

## Author summary

During HIV infection, a reservoir of long-lived latently infected cells is established that persists during antiretroviral therapy (ART) and is the source of virus replication after treatment cessation. A better understanding of when viruses enter the HIV reservoir (reservoir seeding) will aid efforts to target these long-lived HIV infected cells during their establishment. We studied women infected at two different times with two genetically distinct HIV strains (called superinfection), and assessed the genetic relationship between sequences of the HIV strains that circulated throughout infection (pre-ART HIV RNA sequences) and the HIV strains that persisted in reservoir cells (HIV DNA sequences during ART). We estimated when HIV DNA sequences entered the reservoir by identifying the time the most genetically related HIV RNA sequence was detected. In most cases we observed that viruses in the reservoir included both the initial and superinfecting lineages, suggesting reservoir seeding occurs throughout HIV infection. However, the majority of HIV sequences entered the reservoir near the time of ART initiation, suggesting that novel strategies that aim to reduce reservoir size should focus on times immediately prior to ART.

## Introduction

A major obstacle precluding an HIV cure is the establishment of a latent reservoir of HIV-infected cells that persist despite decades of suppressive antiretroviral therapy (ART) [1]. While the majority of HIV-infected cells contain defective proviral genomes, there are cells with full-length intact provirus that are replication competent. Most ART intensification and proviral sequencing studies suggest that there is little to no new infection of host cells while the infected individual takes suppressive ART [2–4]. Persistence of HIV infection is instead driven by infection of long-lived resting CD4+ T cells prior to treatment and clonal proliferation of infected cells [5–7]. Together, this leads to a stable reservoir with an estimated half-life of between 44 months (3.7 years) [8, 9] and 140 months (11.7 years) [10], depending on the assay used to quantify the reservoir. This stable reservoir has necessitated lifelong therapy to prevent viral recrudescence. Understanding when the reservoir is seeded and reservoir dynamics is essential to inform HIV cure approaches.

Multiple studies show that the reservoir is established very early in infection, including a study in an experimental model system of SIV infection in macaques that showed the reservoir is formed in that model prior to detection of viremia in blood [11]. Studies in humans have found that initiating ART early during acute infection reduces reservoir size when compared with later treatment [12, 13], and that early ART may contribute to an increased incidence of post-treatment control [14]. While many studies have shown that early treatment limits reservoir size, questions remain about HIV latent reservoir seeding dynamics. Some studies have suggested that the reservoir is predominantly seeded early in infection, based on data showing that HIV reservoir viruses and viruses that rebound during treatment interruption are often phylogenetically closer to founder virus than viruses present in plasma at ART initiation [15, 16]. Other studies suggest the reservoir is seeded continually throughout infection based on introduction of drug resistance into the reservoir during sub-optimal treatment [17] and on modeling of RNA and DNA sequences throughout infection and subsequent treatment [18]. However, recent studies that compared latent reservoir sequences to longitudinal HIV RNA sequences have reported that the majority of latent reservoir sequences are most closely related to HIV RNA sequences circulating just prior to the initiation of ART [19, 20]. These studies rely mainly on phylogenetic inference from the evolution of a single HIV variant to establish the temporal relationship and seeding dynamics of the latent reservoir.

HIV superinfection represents a unique setting to evaluate the timing of reservoir seeding because the individual is infected initially by one HIV strain and then at a later time with a different and genetically distinct viral strain [21]. Thus, the relative abundance and evolution of the two viral strains that established infection at different times in the reservoir can be assessed to inform phylogenetic inference and help clarify HIV latent reservoir seeding dynamics. In this study we compared longitudinal plasma HIV RNA sequences before treatment to HIV DNA sequences during ART in a cohort of Kenyan HIV superinfected women with known timing of initial infection and superinfection and >6 months of ART-mediated viral suppression. We detected viruses from both the initial and superinfection lineages in the HIV DNA reservoir, confirming that reservoir seeding occurs throughout HIV infection. In addition, we found that the majority of HIV DNA sequences were most closely related to RNA sequences found in plasma at timepoints close to ART initiation.

## Results

### Longitudinal sampling of HIV superinfected women before and after ART initiation

**Fig 1:**
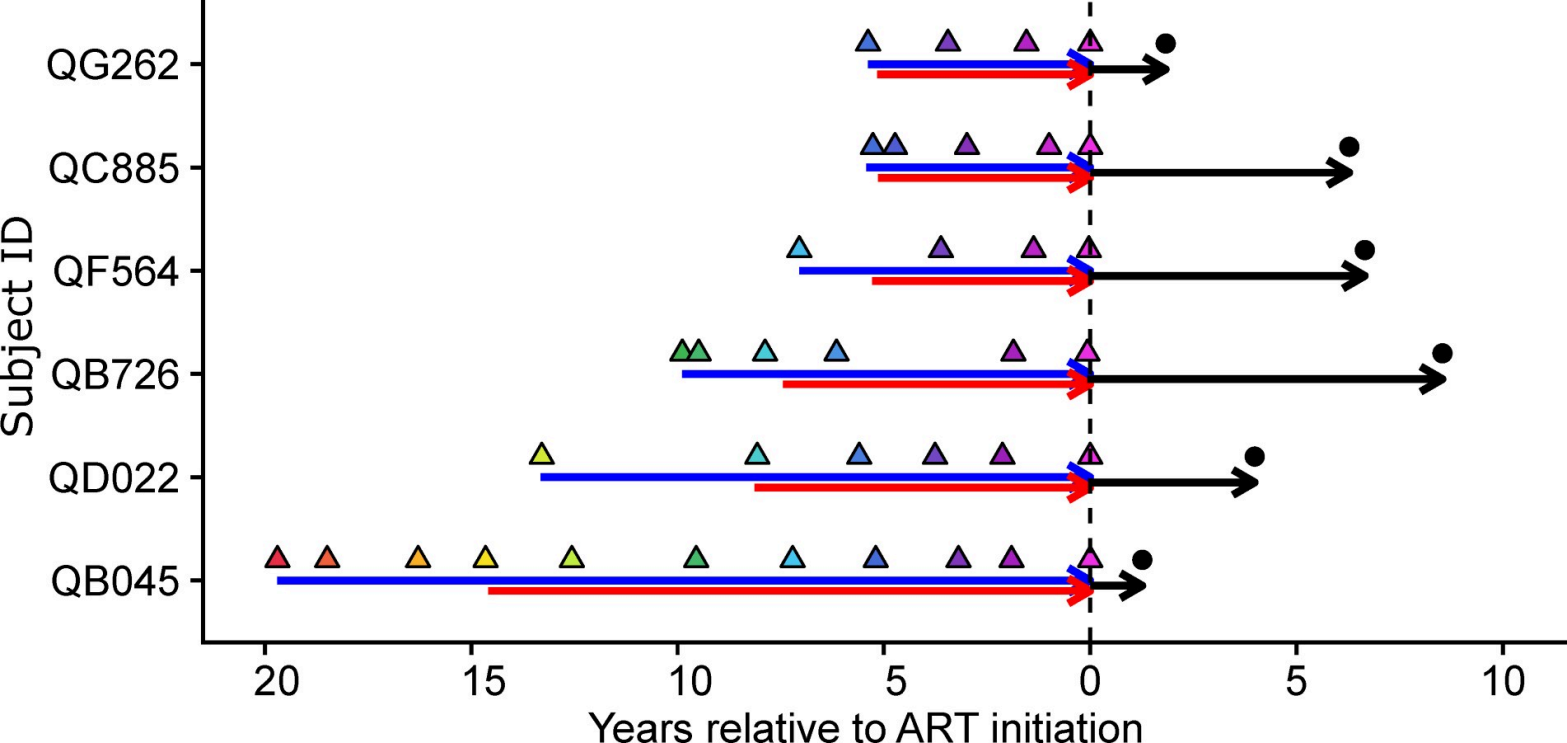
Sampling time points for the 6 superinfection cases. For each subject, horizontal arrows indicate length of initial infection (blue), superinfection (red), and ART (black). Triangles indicate plasma samples for HIV RNA sequencing every ~2 years between initial infection and ART initiation, with colors shaded relative to time prior to ART initiation. Black circles are indicative of the HIV DNA sample time point for each participant.

We previously identified cases of HIV superinfection in a prospective cohort of high-risk women in Mombasa, Kenya and determined the dates of both initial infection and superinfection [22–24]. Six of these cases, which were ART-naïve for >5 years (range 5.4–19.8), were subsequently treated with ART and achieved viral suppression for >6 months (S1A Fig). Among these cases, time between initial infection and superinfection ranged from 0.2–5.2 years (Fig 1). Plasma samples from 0–2 months after the estimated date of initial infection, longitudinal plasma samples collected on average every 2 years (range 0.4–5.2 years) until ART initiation, and a PBMC sample collected after >0.9 years (range 0.9–4.8 years) of suppressive ART were available for next-generation sequencing (NGS) of *gag* and *env* (Fig 1).

### Dynamics of initial and superinfecting HIV RNA lineages prior to ART initiation

**Fig 2:**
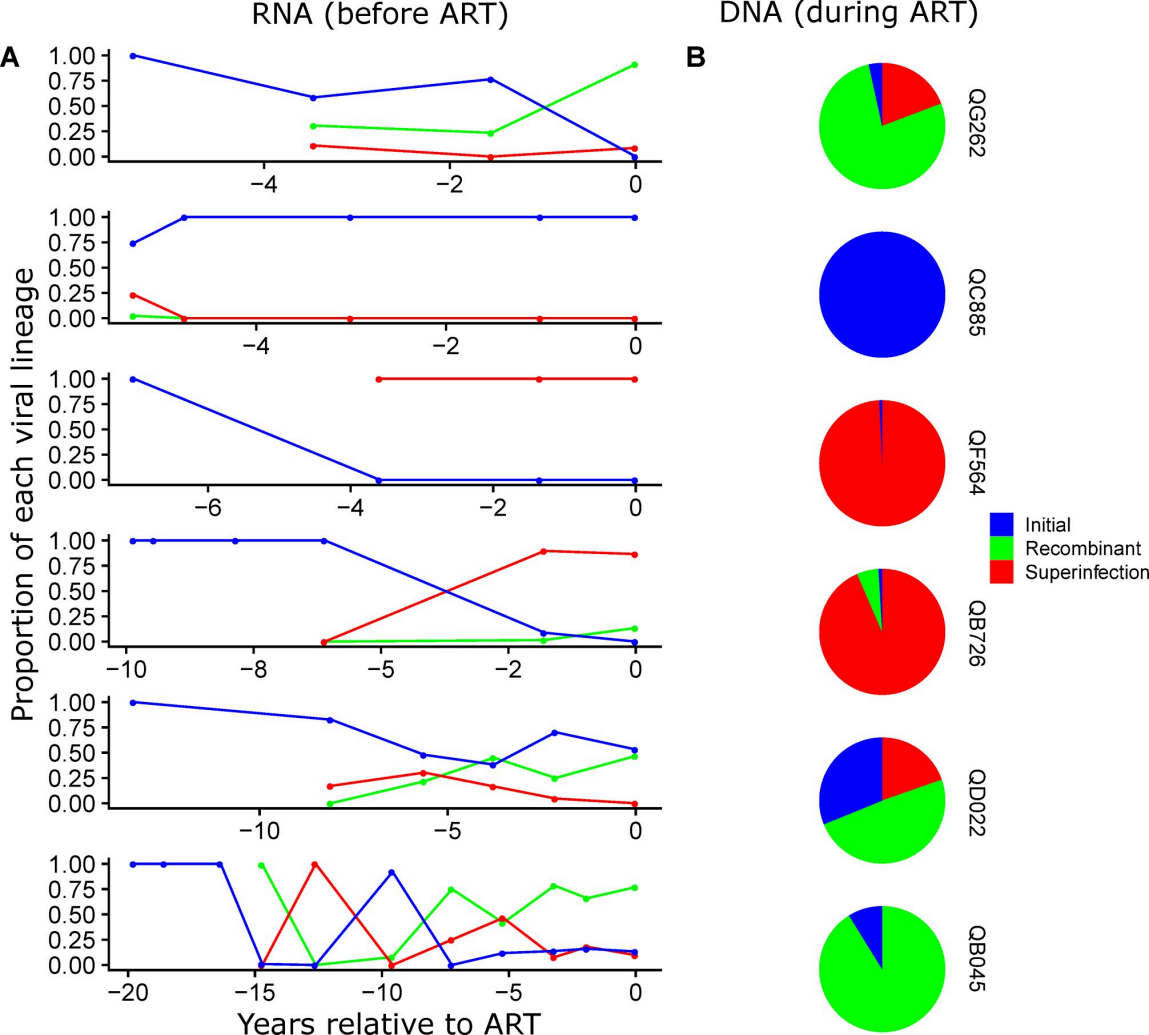
Proportion of *gag* sequences from initial and superinfecting virus lineages prior to and during suppressive ART. (A) Proportions of initial, superinfecting and recombinant virus sequences in HIV *gag* RNA from plasma prior to ART. (B) HIV *gag* DNA from PBMCs collected >6 months after viral suppression on ART. Blue denotes the initial viral variant, red denotes the superinfecting variant, and green denotes within-*gag* recombinants.

HIV RNA sequences in both *gag* and *env* were classified as lineages of the initial, superinfecting or recombinant variants. The relative proportion of each lineage varied over time following superinfection (Fig 2A, S2A Fig). In *gag*, the initial and superinfecting variants co-circulated over time and formed within-region recombinants that became the dominant strain in 3 cases (QG262, QD022, and QB045), while in the other 3 cases either the superinfecting variant (QF564, QB726), or the initial variant (QC885) was dominant following superinfection (Fig 2A). In *env*, there were 3 cases in which the initial and superinfecting variants co-circulated over time (QG262, QB726, QB045), and in 2 of those cases a recombinant virus eventually became dominant (QG262, QB726). In the other 3 cases, the initial variant remained dominant (QC885, QF564, QD022; S2A Fig) and represented more than 93% of plasma RNA sequences at all timepoints. The patterns in *env* did not always reflect the patterns in *gag*, suggesting between-region recombination occurred in some cases.

### Initial and Superinfecting variants in the HIV DNA reservoir during suppressive ART

HIV DNA sequences in *gag* and *env* from PBMCs isolated following >6 months of viral suppression were also classified as lineages of the initial, superinfecting or recombinant variants. In 5 of 6 cases, HIV DNA reservoir *gag* and/or *env* sequences included lineage variants from both the initial and superinfecting virus, and/or within-region recombinants that contained both initial and superinfecting sequences (Fig 2B, S2B Fig). In 1 case, QC885, in which initial lineages remained dominant in HIV RNA after superinfection, only initial virus lineages were present in both *gag* and *env* in the HIV DNA reservoir.

We tested whether the proportion of each virus lineage in the HIV DNA reservoir (Fig 2B and S2B Fig) reflects the dynamics of the variants in the HIV RNA over time (Fig 2A and S2A Fig). To test this, we used a “cumulative” or continuous seeding and decay model (see methods), which assumed that sequences enter the reservoir in proportion to the measured viral load of each variant present at each timepoint, and that specific reservoir sequences decay at a constant rate based on the 44-month half-life estimated from previous studies of decay of replication-competent reservoir cells during suppressive ART [8, 9]. We compared this to a “last timepoint” model, which assumes that the proportion of each virus lineage present in the HIV DNA reservoir is the same as the proportion present in the HIV RNA at the last timepoint assessed prior to ART initiation, as suggested in recent studies [19, 20]. For the cumulative model, we chose a decay rate based on a 44-month half-life from a quantitative viral outgrowth assay (QVOA) which measures only the replication-competent reservoir [8], as opposed to the longer half-life estimate based on decay of total HIV DNA (includes both replication-competent and defective proviruses) [10], because the shorter half-life is a more conservative comparison to the last timepoint model. When compared to our data, the last timepoint model had a significantly lower root mean square (rms) error than the cumulative model for *gag* (Mann-Whitney U test, p = 0.02), indicating better agreement between the last timepoint model and the observed data (S3A Fig). For *env*, the last timepoint model also had a lower median rms error than the cumulative model, but the difference between the models was not significant (p = 0.47), likely due to two cases (QF564 and QB045) in which the lineage proportions in the HIV DNA did not fit either model (S3B Fig). In cases for which a single lineage variant remained dominant throughout infection or when the lineage proportions remained consistent over time, the last timepoint and cumulative model predictions were very similar. Of note, we also assessed the relationships between these models and duration of untreated infection or relative length of time of superinfection, and found no significant correlation (S3C Fig).

### Majority of HIV DNA reservoir sequences phylogenetically cluster with HIV RNA circulating near ART initiation

**Fig 3:**
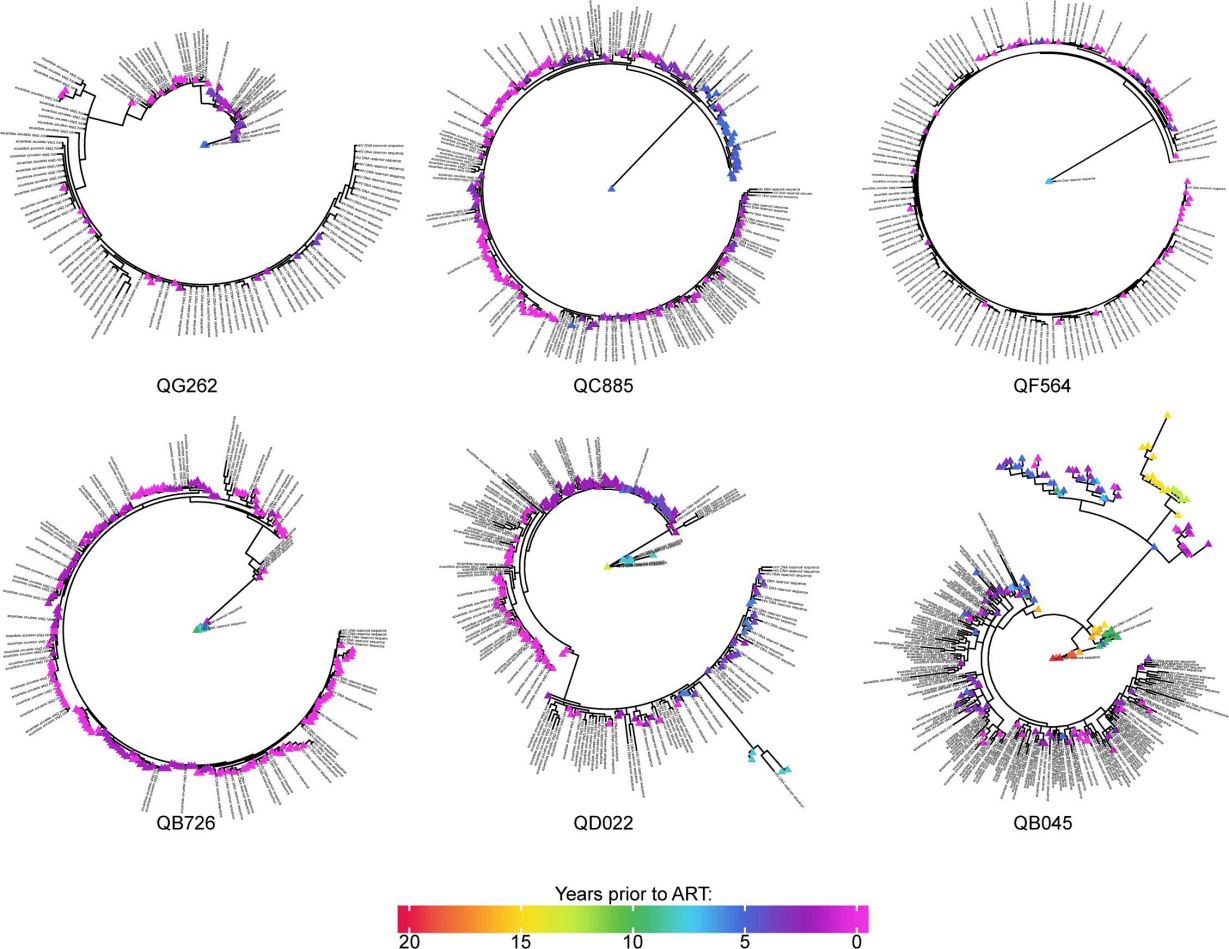
Maximum likelihood phylogenetic trees of HIV *gag* sequences for all 6 subjects. Sequences from pre-ART plasma HIV RNA are indicated by colored triangles according to time prior to ART as shown in color ribbon, and HIV DNA sequences from PBMCs collected during suppressive ART are indicated by black character strings.

For each case of HIV superinfection, we sampled between 50 and 349 unique variants within each gene region in the RNA across all timepoints, and between 28 and 155 unique variants in the DNA at the single timepoint sequenced (S1B Fig & S1C Fig). The number of unique variants we sequenced increased over time, which is expected as evolution causes increases in viral diversity throughout untreated HIV infection. The sequences in the HIV DNA reservoir were interleaved with the complete diversity of the HIV RNA sequences in all cases in *gag* (Fig 3), with some DNA sequences an exact match to the RNA sequences. Similar analysis of *env* also showed HIV DNA and RNA sequences were phylogenetically related (S4 Fig). In all cases, the diversity of HIV DNA sequences reflected the phylogenetic diversity of HIV RNA sequences over time, despite the lower estimate of input HIV DNA, ~500 copies, compared to HIV RNA input, ~4000 copies, for each sample.

**Fig 4:**
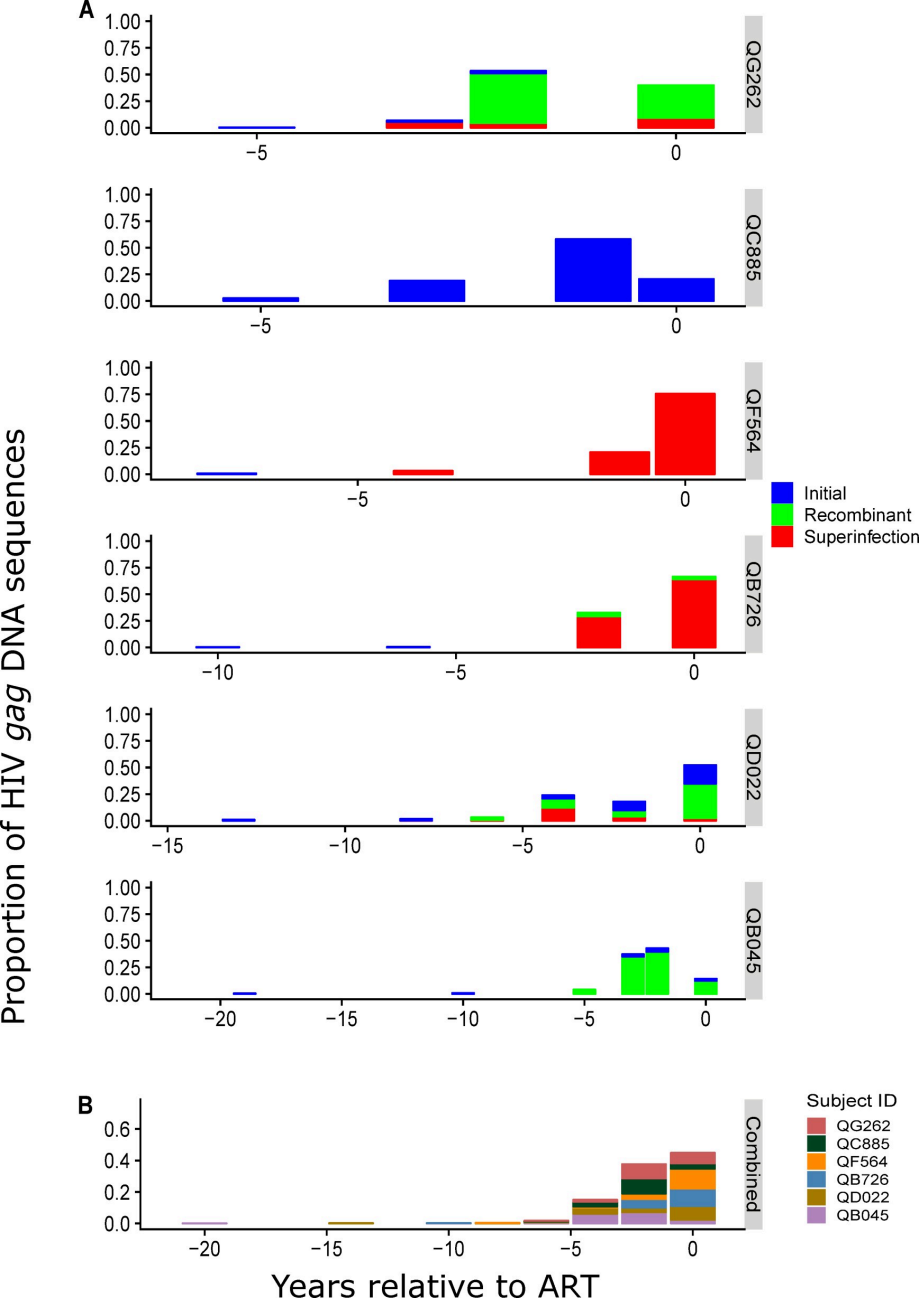
Estimated seeding time of HIV *gag* DNA reservoir sequences in relation to ART initiation. (A) Proportions of the HIV *gag* DNA reservoir sequences at estimated times of seeding prior to ART initiation as determined by the smallest pairwise distance between the HIV *gag* DNA sequences and longitudinal pre-ART HIV *gag* RNA sequences for each subject. Blue denotes virus from the initial infection lineage, red denotes superinfecting virus lineage, and green denotes within-*gag* recombinants. (B) Proportions of HIV *gag* DNA sequences grouped into the nearest 2-year interval from all 6 participants combined. Colors denote individual subjects according to the key.

Pairwise distance analysis between HIV DNA and RNA sequences based on the phylogenetic trees was used to estimate seeding time of each HIV DNA reservoir sequence. The proportion of DNA reservoir sequences that were most similar to the RNA sequences circulating at each timepoint are shown for *gag* in Fig 4 and *env* in S5 Fig. The HIV DNA sequences associated with each timepoint include initial, superinfecting and/or recombinant variants that reflect the composition of the HIV RNA variants from that timepoint (for *gag* compare Figs 2A and 4, for *env* compare S2A Fig and S5 Fig). In most cases, the majority of DNA sequences are most closely related to RNA sequences from times close to ART initiation. For example, for QG262, about 40% of the *gag* DNA sequences were similar to RNA circulating at the time immediately prior to ART and 53% were most closely related to RNA from the timepoint 1.6 years prior to ART, with only minor contributions from early in infection (Fig 4). Data from all 6 individuals combined shows that the majority (median 86%, range 57–99% for *gag;* median 86%, range 69–99% for *env*) of HIV DNA reservoir sequences were most closely related to HIV RNA sequences circulating within 2 years (range 0.0–2.13 years) prior to ART initiation (for *gag* see Fig 4B, for *env* see S5B Fig).

**Fig 5:**
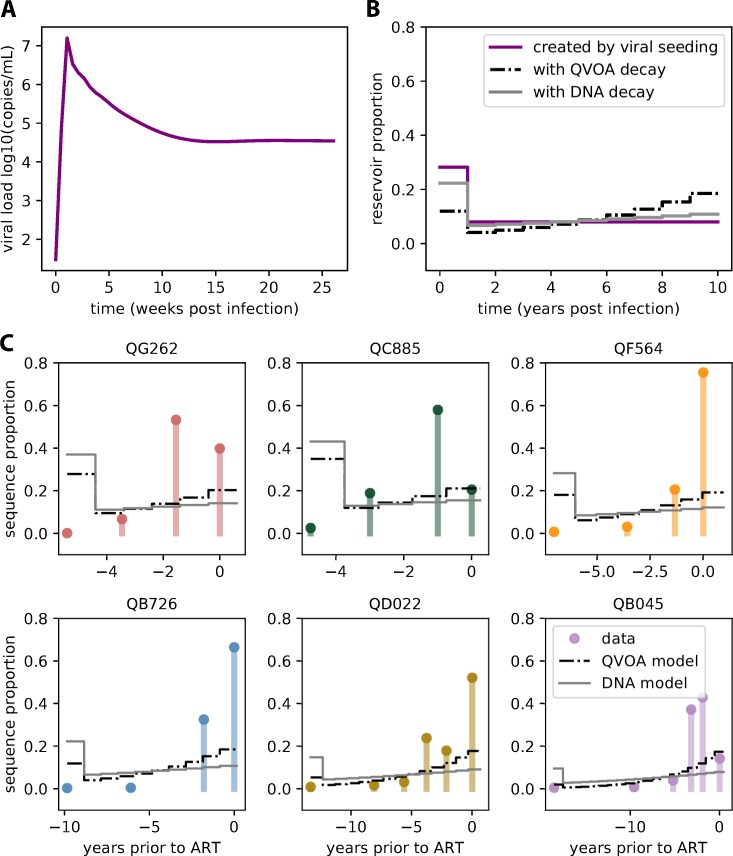
Modeling reservoir creation with constant seeding and decay. (A) A mechanistic mathematical model was used to generate a representative viral load curve. (B) A model of the proportion of cells seeded in the reservoir during each year of infection (purple line) based on the assumption that sequences entered the reservoir in proportion to the viral load dynamics shown in panel A. The model predicted that the highest proportion of reservoir sequences were created during the first year of infection because peak viral load is ~2 logs higher than viral load set-point (see A). We then added a constant decay rate of a 44-month half-life (based on QVOA measurements from Siliciano et al [8]) to the model and plotted the proportion of sequences contributing to the reservoir from each year (dashed black line) over a 10-year simulated infection. This “cumulative” constant seeding and decay model resulted in a bias to sequences created later during infection. This effect is lessened when we used a slower decay rate of 140 month half-life based on total HIV DNA decay [10] (solid grey line). (C) Comparisons of cumulative models to the experimental proportions of *gag* sequences estimated to contribute to the reservoir from each year of infection in 6 individuals. Models based on a half-life of 44 months (dashed black line) or 140 months (grey line) differ with length of infection. Experimental data suggests the reservoir is comprised of a larger number of sequences created near the time of ART initiation than would be expected from models using either clearance half-life. The accuracy of the model to the data was quantified using a non-parametric Kolmogorov-Smirnov test and was significant for all individuals (p<0.01 for all subjects, see S5 Fig) using either half-life model, confirming that the constant seeding and continuous decay model is unlikely to explain the observed data.

Thus, similar to the analysis described above for the proportion of initial and superinfecting variants, the pairwise distance analysis of our data suggest that the composition of the HIV DNA reservoir more closely reflects the HIV RNA sequences circulating at the last timepoint prior to ART rather than the dynamics of HIV RNA sequences over time during untreated infection. In order to formally test this, we created a cumulative model based on the assumption that sequences enter the reservoir in proportion to a representative viral load pattern which included a peak in viremia during acute infection followed by a stable set point viral load (Fig 5A). In Fig 5B we show an example of the proportion of sequences our model predicts would enter the reservoir during each year of untreated infection based on a scenario with 10 years of HIV infection before ART initiation. In this example, ~30% of reservoir sequences are predicted to be created during the first year of infection due to the impact of peak viremia (purple line in Fig 5B). After applying clearance rates based on two different reservoir half-life estimates, the impact from peak viremia decreases, and there is an increase in reservoir sequences created during the years leading up to ART initiation. However, for both of the half-life estimates used, our cumulative models predict that a high proportion of reservoir sequences would originate from primary infection. This effect is slightly diminished in the decay model based on QVOA measurements compared to the total HIV DNA decay model (Fig 5B). We then compared the predictions based on these cumulative models (which are dependent on length of infection prior to ART) to each individual’s observed proportion of reservoir seeded over time (for *gag* see Fig 5C; for *env* see S6 Fig). The results indicate that the cumulative model is inadequate to explain the observed data. In most cases, the model overestimates the proportion of reservoir sequences created during primary infection and peak viremia (Fig 5C and S6B Fig). Moreover, in individuals who had been infected for the longest time periods prior to ART, and even choosing the faster of the half-lives, the model underestimates the proportion of the sequences added to the reservoir during the final 2 years before ART. A Kolmogorov-Smirnov (KS) statistical test indicated that the cumulative models are not supported by our data (S6A and S6C Fig).

**Fig 6:**
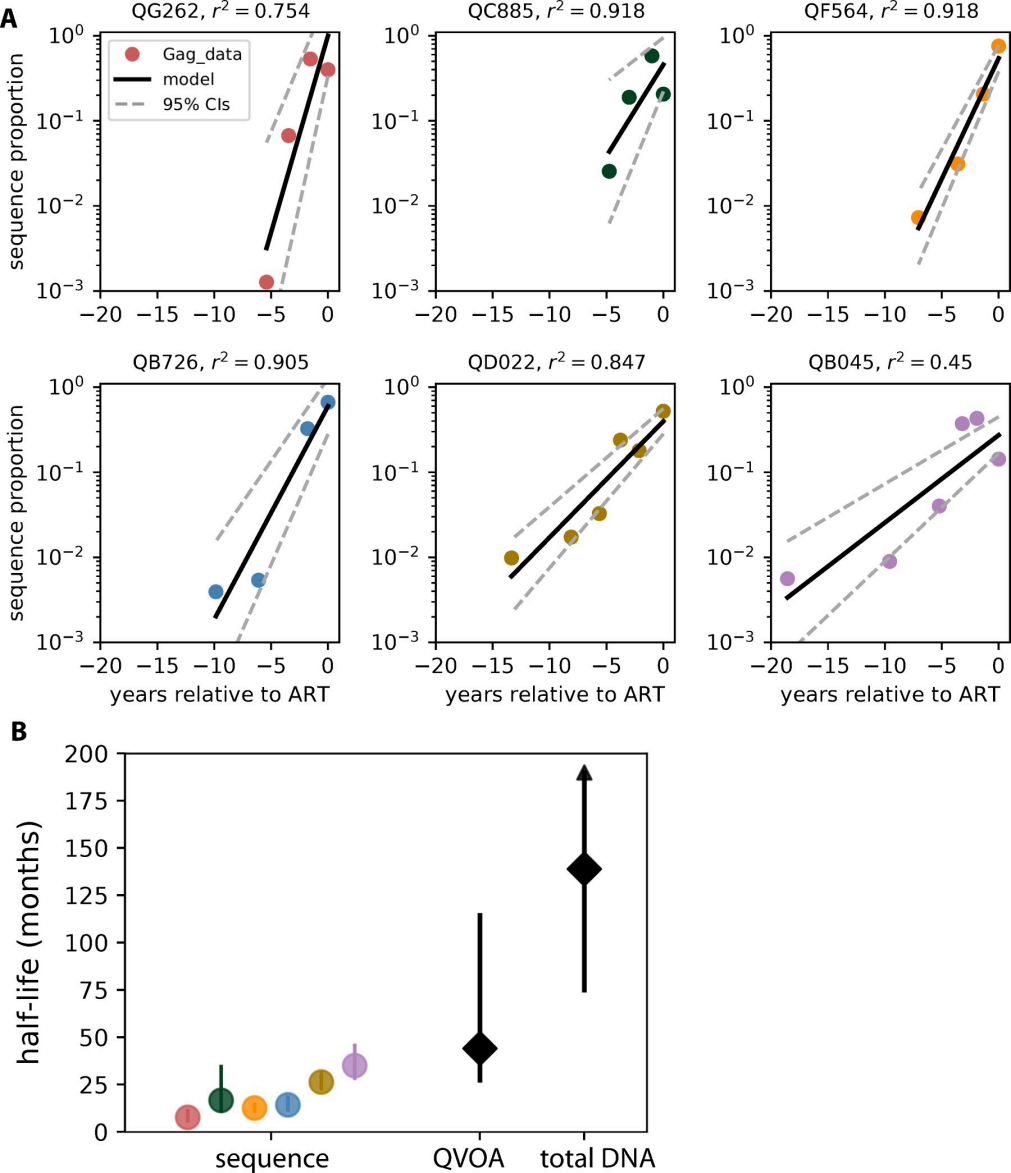
Quantifying *gag* sequence clearance half-life. (A) Log-linear regression plots of relative sequence abundance show an approximately exponential clearance of sequences over time during untreated infection in each of the 6 subjects. (B) Observed total HIV *gag* DNA pre-ART sequence half-lives for each individual based on the log-linear regression are shown: circles (colors by subject as in A) indicate the median estimated half-life and lines show 95% confidence intervals (CIs) associated with each estimate. These are compared to previously reported on-ART reservoir population size half-lives based on replication-competent reservoir decay measured by QVOA [8] and total HIV DNA decay [10]: black diamonds indicate the median estimated half-life and lines show 95% CIs associated with each estimate. Arrows indicate a CI that is inclusive of an infinite half-life (no decay).

We then quantified the clearance rate of reservoir sequences, and compared these to known reservoir half-lives (Fig 6, S7 Fig). Fig 6A shows log-linear regression, based on our *gag* data, to estimate the clearance rate of sequences created over time before ART (identical analysis for *env* sequence clearance shown in S7A Fig). Across individuals, the median half-life was estimated to be 15 (range 7–35) and 35 (range 10–68) months for *gag* and *env*, respectively. Averaging across gene medians results in an estimated half-life of 25 months. For these estimates, we excluded RNA timepoints that were not detected in DNA, however when we include all timepoints and set undetectable levels to a minimum detection limit of 1/500 copies, the estimates are similar: median 13 and 22 months for *gag* and *env*, respectively. Our data shows that each individual’s pre-ART estimated reservoir sequence clearance rate is significantly faster than even the most rapid of the decay rates previously observed during suppressive ART, with no overlap in 95% confidence intervals for any individual for *gag*, and 4/6 with no overlap for *env* (Fig 6B, S7B Fig). Our estimates of the pre-ART half-life, which are based on total HIV DNA sequences, are shorter than either of the reservoir half-lives estimated during suppressive ART: 44 and 140 months based on replication-competent and total HIV DNA [8–10] reservoir measurements, respectively.

## Discussion

In this study we characterized the dynamics of the HIV DNA reservoir in 6 Kenyan women infected with two genetically distinct HIV variants at separate timepoints. We deep sequenced HIV *gag* and *env* RNA from longitudinal plasma collected during primary infection and every ~2 years thereafter until ART initiation. We also sequenced HIV DNA from PBMCs collected after >0.9 years of ART-mediated viral suppression. Our data showed that the HIV DNA reservoir can be comprised of both initial and superinfecting viral variants, excluding the hypothesis that the reservoir is only seeded during early acute infection. In fact, when the initial and superinfecting viruses co-circulated over time during untreated infection, the HIV DNA reservoir was predominantly comprised of superinfecting and/or recombinant virus variants, suggesting that the majority of the HIV DNA reservoir was seeded after superinfection had occurred. In addition, pairwise distances between HIV DNA sequences during ART and longitudinal RNA sequences prior to ART showed that the majority of reservoir sequences were most closely related to HIV RNA sequences from virus circulating in the 2 years prior to ART initiation. These data support previous studies by Brodin et al. and Abrahams et al. which suggested that HIV-infected cells rapidly turn over during untreated infection and that ART-mediated viral suppression creates an environment that favors the persistence of infected cells [19, 20].

Our methods allowed us to interrogate two different regions of the HIV genome, *gag* and *env*. In some cases the viral lineage specific proportions in cell-free HIV RNA differed between *gag* and *env* (compare Fig 2 to S2 Fig), which may reflect recombination that occurred between these regions during active viral replication. We also detected within-region recombination in at least one HIV genomic region in 5 of the 6 cases studied here. The differences in the RNA patterns between and within genomic regions typically reflect differences in the HIV DNA sequences between *gag* and *env–*suggesting that recombinant variants entered the reservoir. However, in two cases (QF564 and QB045), the *env* HIV DNA patterns did not reflect the *env* HIV RNA patterns, which could be a result of missed sequences due to differences in sampling, or clonal proliferation of defective HIV DNA variants not detected in the RNA. Multiple HIV reservoir studies show that the majority of proviruses contain large internal deletions [25, 26], and therefore we cannot rule out that the differences in patterns in HIV DNA sequences between regions is a result of large deletions. We did not perform full-length virus sequencing in this study, which would have allowed us to distinguish full length intact proviruses from defective proviruses, as well as to track full length lineage recombinants over time in RNA and verify their presence in the HIV DNA reservoir. While this is a limitation of our study, independent analysis of both regions supported the conclusion that the majority of observed cells in the HIV reservoir contain sequences that were generated close to the time of ART initiation.

We only sequenced HIV DNA reservoir from a single timepoint after ART-mediated viral suppression, which limits our ability to assess dynamics in the HIV DNA reservoir during ART. However, many studies have shown reservoir stability over time with no sequence evolution and slow decay in population size [19, 27, 28]. Memory CD4+ T cell proliferation is thought to be a critical mechanism for reservoir maintenance over time with individual clones expanding at different rates in terms of size, implying that different clonal proliferation rates may in part determine the sequences detected from a given sample at a single timepoint. Since we did not sequence proviral integration sites, and because the regions we sequenced were short portions of the HIV provirus, we were unable to assess the impact of clonal proliferation. In addition, it is well established that the majority of proviruses present in PBMCs during ART are replication incompetent [26]. We sequenced two ~500bp regions of HIV DNA present in PBMCs, which did not allow us to distinguish between defective and replication competent virus in the reservoir. Presumably, a majority of our sampled HIV DNA sequences have incurred lethal mutations or deletions, while the majority of HIV RNA sequences were likely from intact viral genomes. It is thought that cells infected with replication-competent virus or defective virus could have different decay rates [29], and therefore it is uncertain whether our conclusions can be extrapolated to the replication-competent HIV reservoir.

For our modeling, we chose to use the 44-month half-life based on replication-competent assay measurements during ART (as opposed to the longer 140-month half-life estimate for total HIV DNA reservoir decay) to show that our observed data appeared more similar to the last timepoint model even with the shorter half-life, despite the fact that our sequencing assessed total HIV DNA. Our half-life estimates differ slightly between *gag* and *env*, but estimates from both regions suggest that older sequences are depleted in the reservoir much more rapidly during untreated infection than would be expected from the decay rates estimated during ART. The average of our sequence half-life estimates is ~25 months, which is longer than the 8 month half-life estimated by Brodin et al [19]. This may be due to different sampling schemes, as Brodin et al had finer sequence resolution during the two years prior to ART initiation. Additionally, it has been observed that viral loads may be increased by superinfection [30]. We did not include a second peak viremia at the time of superinfection in our model, which may have resulted in an underestimate of reservoir seeding at the time of superinfection. However, we found no significant correlation between model scores and the time of superinfection or duration of infection pre-ART (S3C Fig, Pearson correlation, p>0.1).

It has been suggested that HIV reservoir seeding is dynamic and that initiation of ART may drive reservoir creation and/or decrease reservoir clearance [20]. In agreement with this observation, we observed a faster clearance rate of reservoir sequences prior to ART than previously observed clearance rates of the total reservoir during ART. There are several mechanistic hypotheses that could explain the discrepancy. Reservoir clearance rate represents the sum of several mechanistic processes including new viral infections, transition between effector and memory cells, clonal proliferation, and cell death due to HIV reactivation and/or immunologic clearance. These component rates could change over time during the course of infection, or at ART initiation. For example, upon ART initiation there could be an increase in the transition from effector to memory HIV-infected CD4+ T cells [19, 20], and/or clonal proliferation of reservoir cells could increase. Future experiments could help specify the mechanism by combining sequence proportions with absolute measurements of reservoir size (which could distinguish increased birth from decreased death) and/or observations of sequence clonality (which could distinguish increased seeding from increased proliferation).

Of importance to clinical studies, ours and the previous studies [19, 20] collectively provide a body of evidence suggesting that cure strategies that target HIV infected cells generated before or coincident with ART initiation may disproportionately reduce HIV reservoir size, increasing the likelihood of post-treatment control and a functional cure.

## Methods

### Study population

Cases of HIV superinfection were identified within a cohort of women at high risk of HIV infection in Mombasa, Kenya, and timing of both initial infection and superinfection were estimated as described previously [22–24]. Cases of HIV superinfection were included in this sub-study based upon the following criteria: 1) follow-up includes time before ART (note: this cohort was initiated before ART was available in this setting), 2) follow-up includes time after ART initiation, 3) longitudinal plasma samples available between initial infection and ART initiation with a mean of 2 years between sample timepoints, 4) PBMC sample available following ≥6 months of ART mediated viral suppression (plasma HIV RNA <150 copies/mL with at most 1 viral blip <1000 copies/mL).

### Ethics statement

This study was approved by the ethical review committees at Kenyatta National Hospital, the University of Washington, and the Fred Hutchinson Cancer Research Center. All participants were adults and provided written informed consent for participation.

### HIV Genome amplification and sequencing

HIV virions were isolated using the μMACS VitalVirus HIV Isolation Kit (Miltenyi Biotec) from 10–400μl of plasma to enable isolation of an estimated ~4,000 viral particles based on the Gen-Probe Viral load assay (Hologic). RT-PCR was performed in duplicate to amplify ~500 base pair regions of *gag* and *env*, as previously described[24]. HIV DNA was isolated from PBMCs by Qiagen DNA Midi Kit (Qiagen). Droplet-digital PCR was performed on an aliquot of the purified DNA to determine HIV DNA genome copy number in each sample, and an estimated 125 HIV DNA genomes were input into quadruplicate PCR reactions to amplify *gag* and *env* regions (as above) from ~500 HIV DNA genomes in total. Nextera adaptors were added by 10 cycles of PCR using Kappa HiFi Polymerase. PCR products were cleaned by AMPure XP purification beads with 1:1 sample to bead ratio, quantified by Qubit dsDNA HS kit and equal amounts pooled into 20μl for barcode addition. Illumina barcodes were then added by PCR ligation to uniquely identify each sample, cleaned by AMPure XP purification beads, and pooled. The pooled library was gel isolated using a Zymoclean kit and sequenced on an Illumina Miseq using 2x300 bp paired-end reads. Sequences are available through the NIH Sequence Read Archive: https://www.ncbi.nlm.nih.gov/bioproject/PRJNA566147.

### Bioinformatic pipeline

Demultiplexing, denoising, and amplicon sequence variant clustering was performed using DADA2[31], identical sequences were collapsed, and read counts of unique variants were determined. Common lab HIV sequences and cross-contamination were removed from the analysis by BLAST against a local database containing patient-specific sequences from previous studies [22–24]. Sequences with an abundance of less than 0.5% were also removed. Consensus sequences of the initial and superinfecting lineage variants were made from sequencing data from previous studies [22–24] and used in the RAPR tool on the LANL website to enable classification of sequences from this study as initial, superinfecting, or recombinant lineage variants. The initial, superinfecting, and recombinant lineage groups identified by RAPR were used to form lineage-specific consensus sequences via the Biopython (https://biopython.org/) Python package. Individual sequences were compared against the representative RAPR-identified lineage consensus sequence to identify hypermutant variants in the context of a given lineage using a custom Python script similar to Brodin et al [19]. Variants containing deletions of >50 base pairs, were filtered out of the sequencing data to control for the noise they contributed to the phylogenetic methods. Proportions of each variant were determined based on read count. Nucleotide diversity (pi), as measured by the DendroPy Python package, was compared between sampled DNA and RNA sequences to verify that sampling accounted for a comparable degree of the true variance in each virus population.

### Phylogenetic and pairwise distance analysis

Phylogenetic trees were constructed using the maximum likelihood method with the R package phangorn [32]. Trees were then annotated using the R package ggtree [33]. Pairwise distances between sequences were computed using the R package ape [34], and DNA sequences were matched to the RNA sequence with the smallest pairwise distance. Primary analysis was done including hypermutated sequences. Secondary analysis excluding hypermutants showed similar results.

### Mathematical modeling of HIV reservoir creation and persistence

We used a naïve model of continuous reservoir seeding and constant reservoir decay, called the “cumulative” model, and tested whether observed HIV DNA sequences in the reservoir could be explained by this model. In the analysis of the initial and superinfecting viral lineages, we created a cumulative model with longitudinal measured viral load data for each variant. However, because peak viremia may have been missed in our measured viral load data due to sampling, for our pairwise distance analysis we created a cumulative model with viral load kinetics based on a typical parameterization and mathematical model of HIV primary infection [35] that included representative HIV primary infection viral load kinetics (see Robb et al. NEJM 2016 [36]). This model is a set of ordinary differential equations describing the time dynamics of the concentrations (per μL) of cells and virus over time. It included susceptible target cells *S*, actively and latently infected cells that are productively infected (P) and produce viable virus *A*_*P*_, *L*_*P*_, as well as actively and latently infected cells that are unproductively (U) infected and cannot produce viable virus *A*_*U*_,*L*_*U*_, virus *V*, and an adaptive immune compartment *E*. This was expressed as:

$$\dot{S}=\alpha_{S}-\delta_{S}S-\beta SV$$

$${\dot{A}}_{P}=\tau \beta SV-\delta_{I}A_{P}-\kappa A_{P}E$$

$${\dot{A}}_{U}=(1-\tau )\beta SV-\delta_{I}A_{U}-\kappa A_{U}E$$ (1)

$${\dot{L}}_{P}=\lambda \tau \beta SV$$

$${\dot{L}}_{U}=\lambda (1-\tau )\beta SV$$

$$\dot{E}=\alpha_{E}+\omega (A_{P}+A_{U})E/(1+E/E_{50})-\delta_{E}E$$

$$\dot{V}=\pi A_{P}-\gamma V-\beta SV,$$

where derivative in time was indicated by over-dot notation, $\dot{x}{=\partial }_{t}x$. The representative parameter set used susceptible cell creation rate *α*_*S*_ = 70 cells μL^-1^ day^-1^ and death rate *δ*_*S*_ = 0.2 day^-1^, viral infectivity *β* = 10^−4^ μL cells^-1^ day^-1^, probability of productively infectious virions *τ* = 0.05, probability of latency *λ* = 10^−4^, actively infected cell death rate *δ*_*I*_ = 0.8 day^-1^, viral burst size *π* = 5x10^4^ virions cells^-1^, viral clearance rate *γ* = 23 day^-1^, initial adaptive precursor frequency *α*_*E*_ = 10^−4^ cells μL^-1^ day^-1^, adaptive immune killing rate *κ* = 0.3 μL cells^-1^ day^-1^, adaptive immune recruitment rate *ω* = 1.6 μL cells^-1^ day^-1^, adaptive immune clearance rate *δ*_*E*_ = 0.002 day^-1^, and adaptive immune 50% saturation constant *E*_50_ = 250 cells μL^-1^. Latent cells were only created in this part of the model, and grouped by their year of creation. The decay of latently infected cells was implemented separately as follows: We considered each year of reservoir seeding its own sequence group. Next, we allowed each of these sequence groups to decay exponentially with a typical HIV reservoir decay rate (e.g. *τ*_{1/2}_ = 44 months, for the QVOA+ half-life identified by Siliciano et al.[8]). We therefore calculated the proportion of sequences remaining from each creation-year interval after an arbitrary number of years of infection and ART. Note, whether viral load was imputed directly from data or modeled, reservoir creation was assumed to be proportional to viral load. The proportionality (see *λ* in Eq 1) was assumed to be constant over time. Further, in this naïve model, we assumed proportions of sequences did not change after initiation of ART: while on ART, the total reservoir size decreased, but the proportional makeup did not change.

### Comparison of modeled and observed reservoir proportions

We compared the cumulative model estimates of sequence proportions to observed data using the two sample Kolmogorov-Smirnov statistic (KS), which tests the probability that two data sets arose from the same underlying distribution.

### Calculating sequence half-lives during chronic infection

Using the proportions of sequences, we fit a log-linear model to each individual and for each gene to extract a sequence half-life and associated 95% confidence intervals. That is, for each individual *i*, and gene *g*, we calculated the proportions from each RNA timepoint *t*_*RNA*_ relative to the initiation of ART, which we expressed as *P*_*i*_(*t*_*RNA*_,*g*). Then we fit a model ln*P*_*i*_(*t*_*RNA*_,*g*) = *b*−*m*⋅*t*_*RNA*_, estimated the slope *m*, converted this to include dimensions of per month, and calculated the half-life *τ*_{1/2}_ = ln2/*m*. Implementation used the SciPy library (curve_fit). We excluded RNA timepoints that were not quantified in HIV DNA. However, results were not changed substantially when we repeated the analysis assuming a lower detection limit where any RNA timepoint with no assigned HIV DNA sequences was set to a detection limit of *P*_*i*_(*t*_*RNA*_,*g*) = 1/500.

## Supporting information

S1 Fig Viral load patterns and numbers of unique HIV variants sequenced throughout infection. (A) Viral loads patterns (log HIV RNA copies per milliliter) prior to and after initiation of ART. Line colors denote individual subjects according to the key. Arrows of the same color denote the time of sampling for HIV DNA sequencing for each subject. (B) The number of unique HIV *gag* variants sequenced throughout infection according to each subject ID by color. (C) The number of unique HIV *env* variants sequenced throughout infection according to each subject ID by color.
(TIF)

S2 Fig Proportion of *env* sequences from initial and superinfecting virus lineages prior to and during suppressive ART. (A) Proportions of initial, superinfecting and recombinant virus sequences in HIV *env* RNA from plasma prior to ART. (B) HIV *env* DNA from PBMCs collected >6 months after viral suppression on ART. Blue denotes the initial viral variant, red denotes the superinfecting variant, and green denotes within-*env* recombinants.
(TIF)

S3 Fig Proportion of virus lineages in HIV DNA reservoir as predicted by the cumulative and last timepoint models compared to the proportions observed in each of the 6 subjects. (A) Proportions of the HIV *gag* DNA sequences classified as initial (Init), superinfecting (Super) or recombinant (Recom) virus lineages based on observed data (black diamonds), the cumulative model (teal lines) or the last timepoint model (purple lines) of each individual subject ID in graphs on left. Comparison of the root mean squared (rms) error between each model and the individual subject data was statistically significant for *gag* (p = 0.023, Mann-Whitney U test) shown in panel on right. (B) Proportions of HIV *env* DNA virus lineages for each individual subject ID in graphs on left (details as in A). Panel on the right compares rms errors for the two models based on *env* data; difference between the models did not reach statistical significance (p = 0.47, Mann-Whitney U test). (C) Comparisons of the rms error for each model with duration of infection pre-ART (left panel), the time between initial infection and superinfection (middle panel), and the time between superinfection and ART initiation (right panel). No correlations were observed between these clinical variables and model fit (p-values shown are calculated using Pearson correlation).
(TIF)

S4 Fig Maximum likelihood phylogenetic trees of HIV *env* sequences for all 6 subjects. Sequences from pre-ART plasma HIV RNA are indicated by colored triangles according to time prior to ART as shown in the color ribbon, and HIV DNA sequences from PBMCs collected during suppressive ART are indicated by black character strings. Note that a single HIV *env* DNA variant of the superinfection lineage was removed from the QD022 tree shown, as the superinfection lineage was not detected in the RNA for this region and due to the phylogenetic distance, including this single HIV DNA variant obscures the ability to see the phylogenetic relationship between the other HIV DNA and RNA *env* sequences shown here.
(TIF)

S5 Fig Estimated seeding time of HIV *env* DNA reservoir sequences in relation to ART initiation. (A) Proportions of HIV *env* DNA reservoir sequences at estimated times of seeding prior to ART as determined by the smallest pairwise distance between HIV *env* DNA reservoir sequences and longitudinal pre-ART HIV *env* RNA sequences for each subject. Blue denotes virus from the initial infection lineage, red denotes superinfecting virus lineage, and green denotes within-*env* recombinants. (B) Proportions of HIV *env* DNA sequences grouped into the nearest 2 year interval from all 6 participants combined. Colors denote individual subjects according to the key.
(TIF)

S6 Fig Comparisons of observed proportions of HIV DNA reservoir sequences seeded over time with proportions predicted by cumulative models. (A) Observed cumulative proportion of HIV *gag* sequences that contribute to the HIV DNA reservoir over time prior to ART (circles connected by dashed lines colored according to individual subject ID). Proportions of HIV *gag* sequences that contribute to the reservoir over time as predicted by the cumulative models using on-ART estimated decay rates of 44-month half-life based on QVOA measurements (black dashed line) and the 140-month half-life based on total HIV DNA measurements (grey solid line). Comparison of the models to the observed data was performed using a non-parametric Kolmogorov-Smirnov test and the difference between the models and the observed data was significant (p<0.01) for all individuals using either half-life model, confirming that the constant seeding and continuous decay model is unlikely to explain the observed data. (B) Comparisons of cumulative models to the experimental proportions of *env* sequences estimated to contribute to the reservoir from each year of infection in 6 individuals. Models based on a half-life of 44 months (dashed black line) or 140 months (grey line) differ with length of infection. Experimental data suggests the reservoir is comprised of a larger number of sequences created near the time of ART initiation than would be expected from models using either clearance half-life. (C) Observed cumulative proportion of *env* sequences that contribute to the HIV DNA reservoir over time prior to ART are compared to proportions of *env* sequences predicted by the cumulative models (graph details as in panel A).
(TIF)

S7 Fig Quantifying *env* sequence clearance half-life. (A) Log-linear regression plots of relative sequence abundance show clearance of *env* sequences over time during untreated infection in each of the 6 subjects. (B) Observed total HIV *env* DNA pre-ART sequence half-lives for each individual based on the log-linear regressions are shown: circles (color by subject ID as in A) indicate the median estimated half-life and lines show 95% confidence intervals (CIs) associated with each estimate. These are compared to previously reported on-ART reservoir population size half-lives based on replication-competent reservoir decay measured by QVOA [8] and total HIV DNA decay [10]: black diamonds indicate the median estimated half-life and lines show 95% CIs associated with each estimate. Arrows indicate a CI that is inclusive of an infinite half-life (no decay).
(TIF)
