# Supplementary figures and images for: Dynamics of HIV DNA reservoir seeding in a cohort of superinfected Kenyan women
Source: PLoS Pathog. 2020 Feb 5;16(2):e1008286. doi: 10.1371/journal.ppat.1008286 (PMC7028291; doi:10.1371/journal.ppat.1008286)

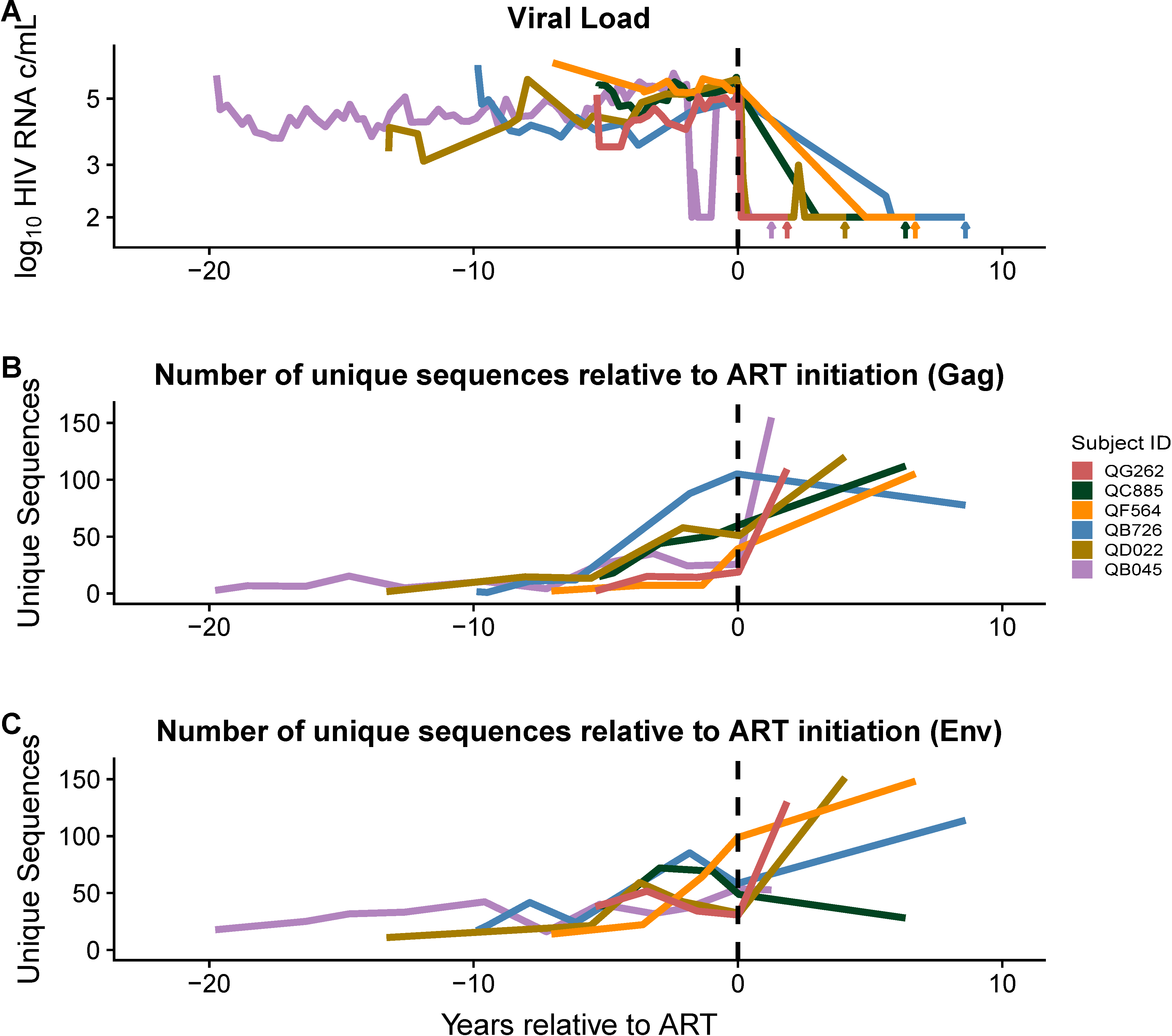

Supplement: S1 Fig — (A) Viral loads patterns (log HIV RNA copies per milliliter) prior to and after initiation of ART. Line colors denote individual subjects according to the key. Arrows of the same color denote the time of sampling for HIV DNA sequencing for each subject. (B) The number of unique HIV gag variants sequenced throughout infection according to each subject ID by color. (C) The number of unique HIV env variants sequenced throughout infection according to each subject ID by color. (TIF) [file ppat.1008286.s001.tif]

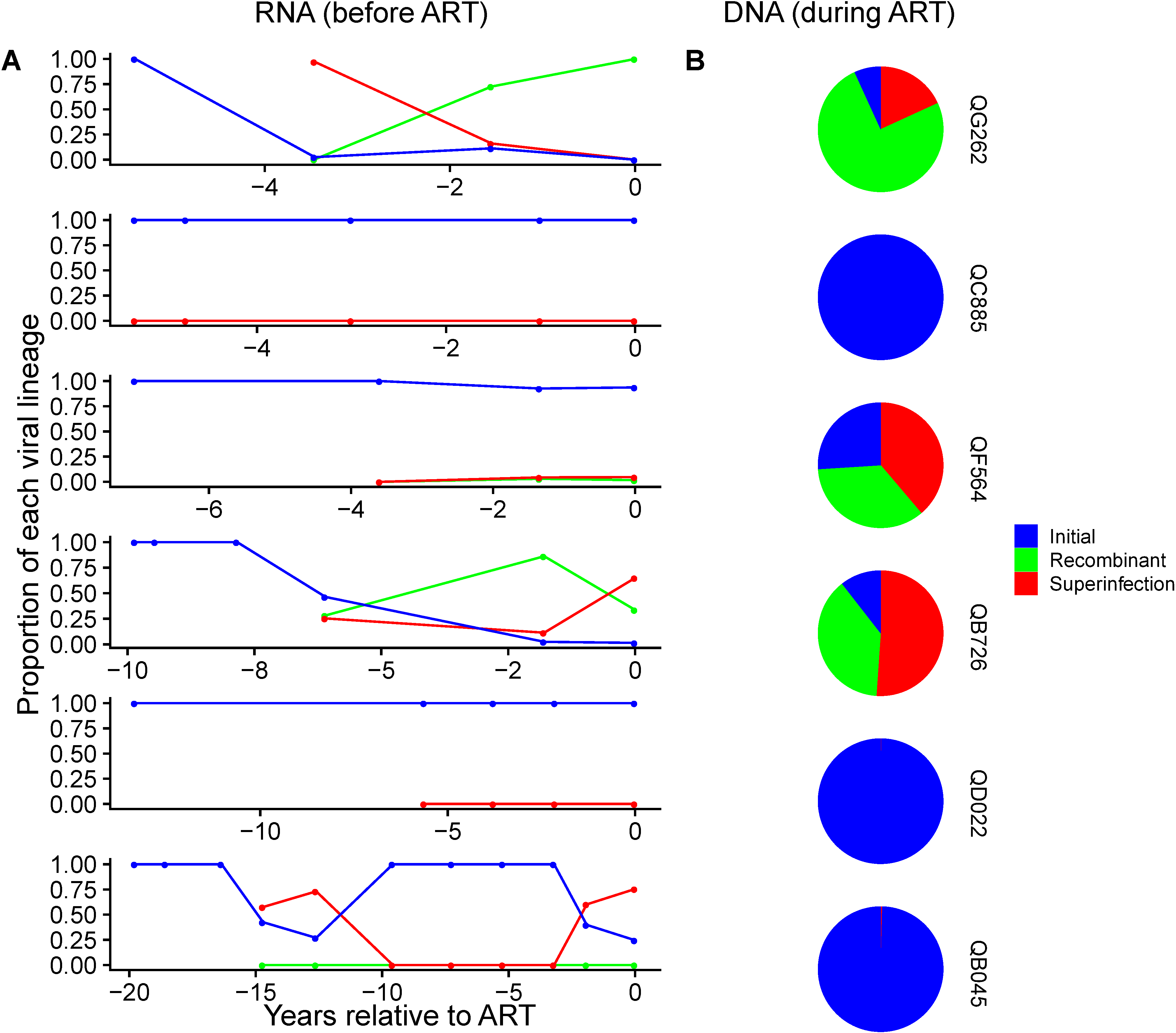

Supplement: S2 Fig — (A) Proportions of initial, superinfecting and recombinant virus sequences in HIV env RNA from plasma prior to ART. (B) HIV env DNA from PBMCs collected >6 months after viral suppression on ART. Blue denotes the initial viral variant, red denotes the superinfecting variant, and green denotes within-env recombinants. (TIF) [file ppat.1008286.s002.tif]

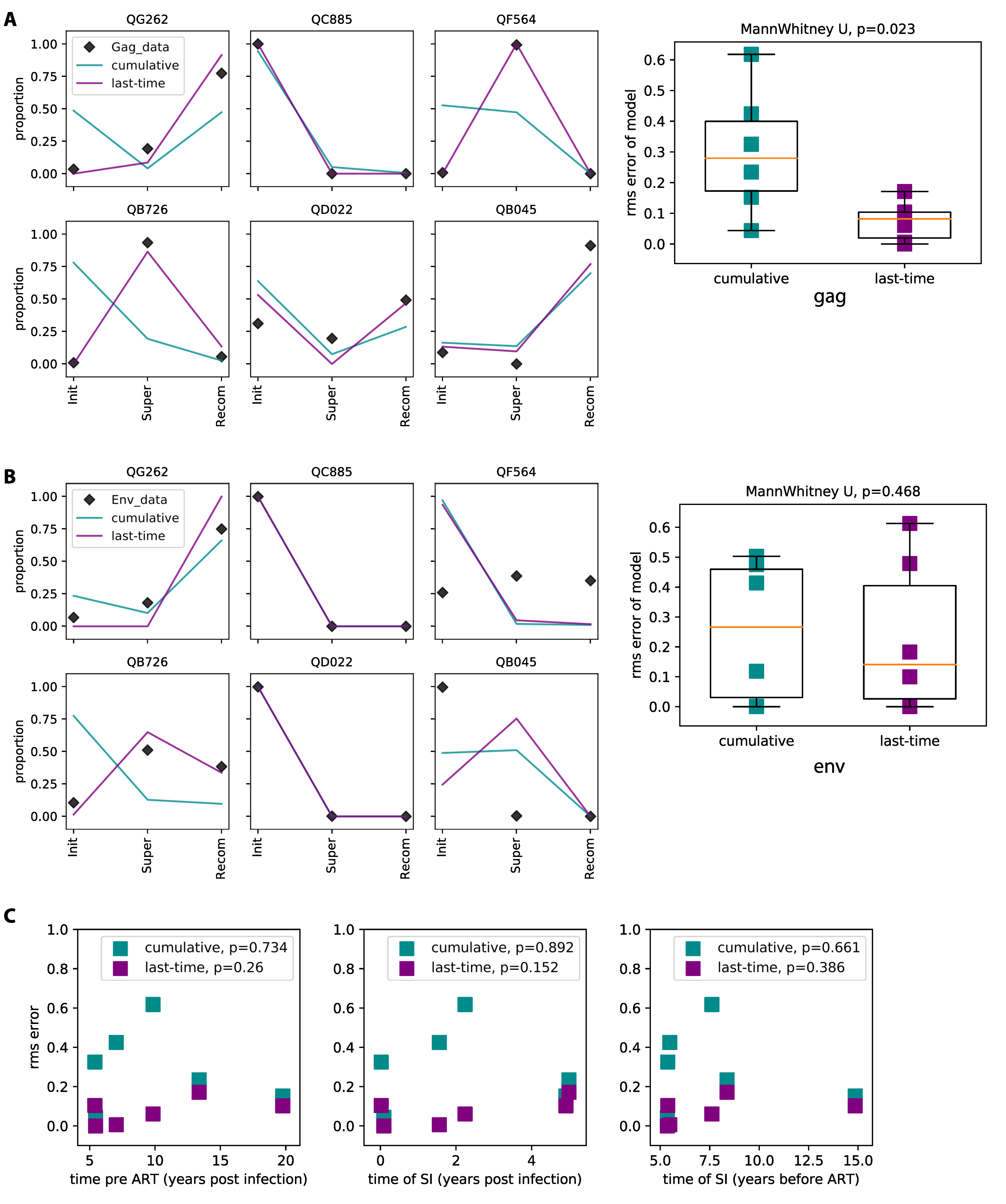

Supplement: S3 Fig — (A) Proportions of the HIV gag DNA sequences classified as initial (Init), superinfecting (Super) or recombinant (Recom) virus lineages based on observed data (black diamonds), the cumulative model (teal lines) or the last timepoint model (purple lines) of each individual subject ID in graphs on left. Comparison of the root mean squared (rms) error between each model and the individual subject data was statistically significant for gag (p = 0.023, Mann-Whitney U test) shown in panel on right. (B) Proportions of HIV env DNA virus lineages for each individual subject ID in graphs on left (details as in A). Panel on the right compares rms errors for the two models based on env data; difference between the models did not reach statistical significance (p = 0.47, Mann-Whitney U test). (C) Comparisons of the rms error for each model with duration of infection pre-ART (left panel), the time between initial infection and superinfection (middle panel), and the time between superinfection and ART initiation (right panel). No correlations were observed between these clinical variables and model fit (p-values shown are calculated using Pearson correlation). (TIF) [file ppat.1008286.s003.tif]

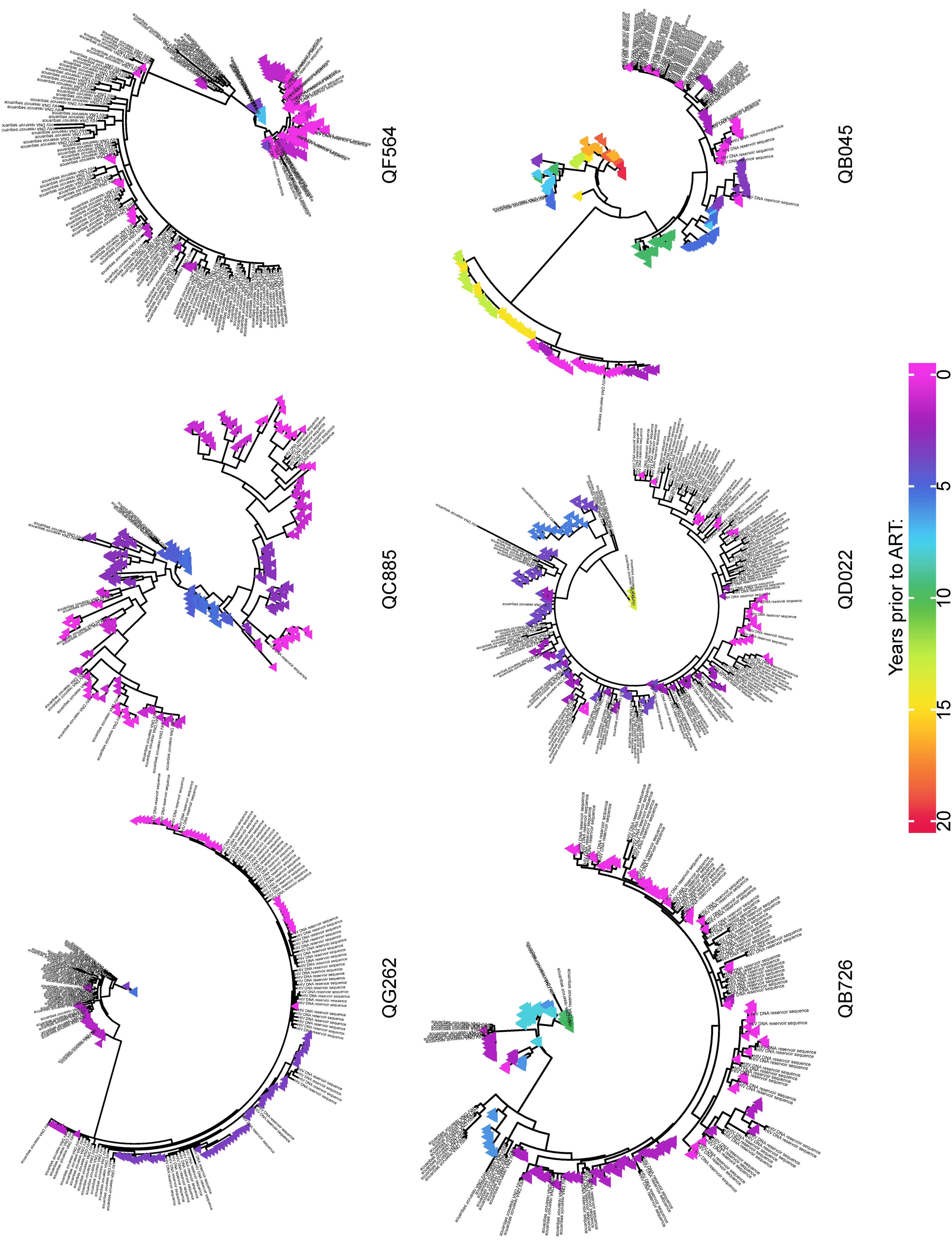

Supplement: S4 Fig — Sequences from pre-ART plasma HIV RNA are indicated by colored triangles according to time prior to ART as shown in the color ribbon, and HIV DNA sequences from PBMCs collected during suppressive ART are indicated by black character strings. Note that a single HIV env DNA variant of the superinfection lineage was removed from the QD022 tree shown, as the superinfection lineage was not detected in the RNA for this region and due to the phylogenetic distance, including this single HIV DNA variant obscures the ability to see the phylogenetic relationship between the other HIV DNA and RNA env sequences shown here. (TIF) [file ppat.1008286.s004.tif]

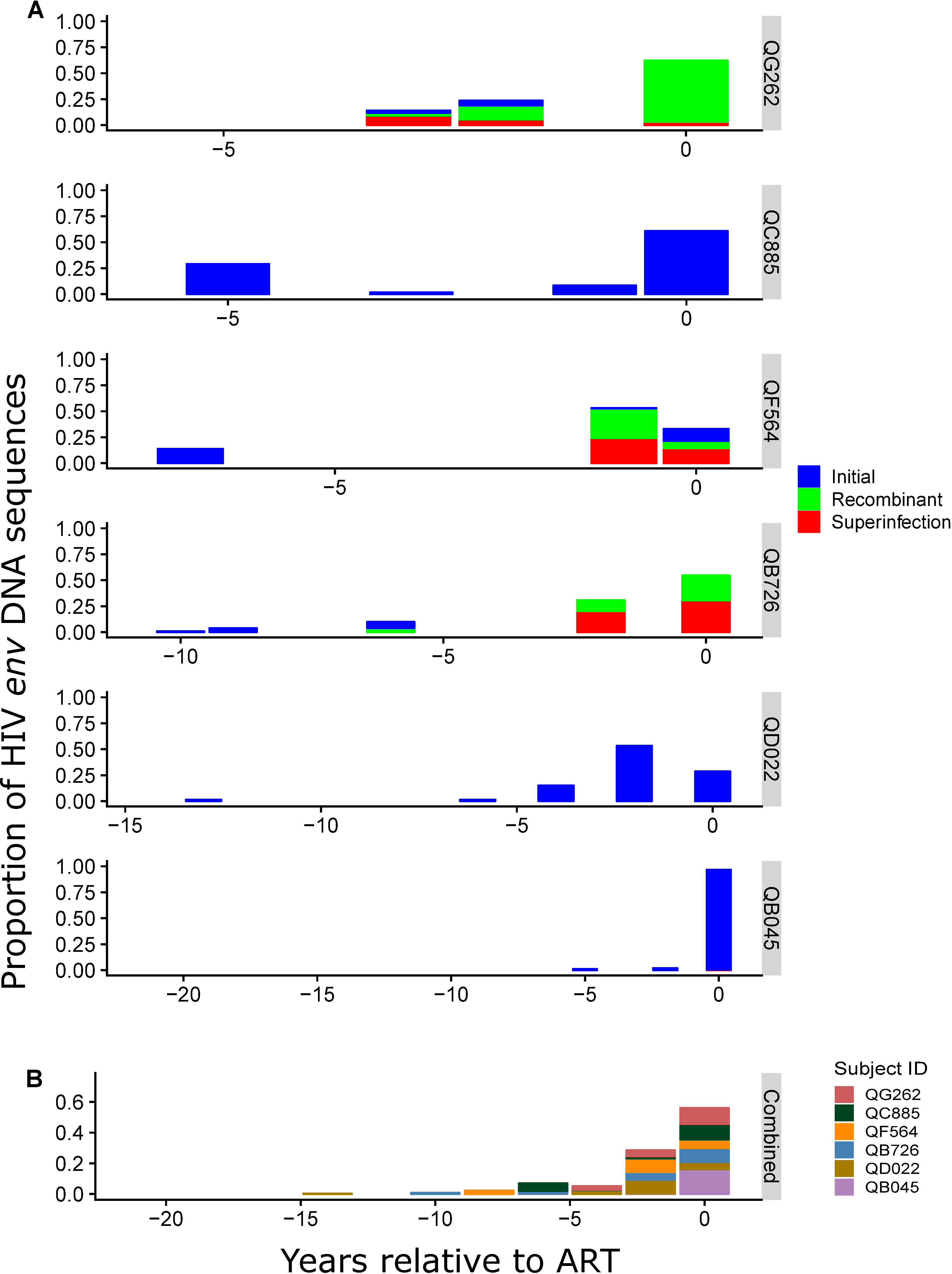

Supplement: S5 Fig — (A) Proportions of HIV env DNA reservoir sequences at estimated times of seeding prior to ART as determined by the smallest pairwise distance between HIV env DNA reservoir sequences and longitudinal pre-ART HIV env RNA sequences for each subject. Blue denotes virus from the initial infection lineage, red denotes superinfecting virus lineage, and green denotes within-env recombinants. (B) Proportions of HIV env DNA sequences grouped into the nearest 2 year interval from all 6 participants combined. Colors denote individual subjects according to the key. (TIF) [file ppat.1008286.s005.tif]

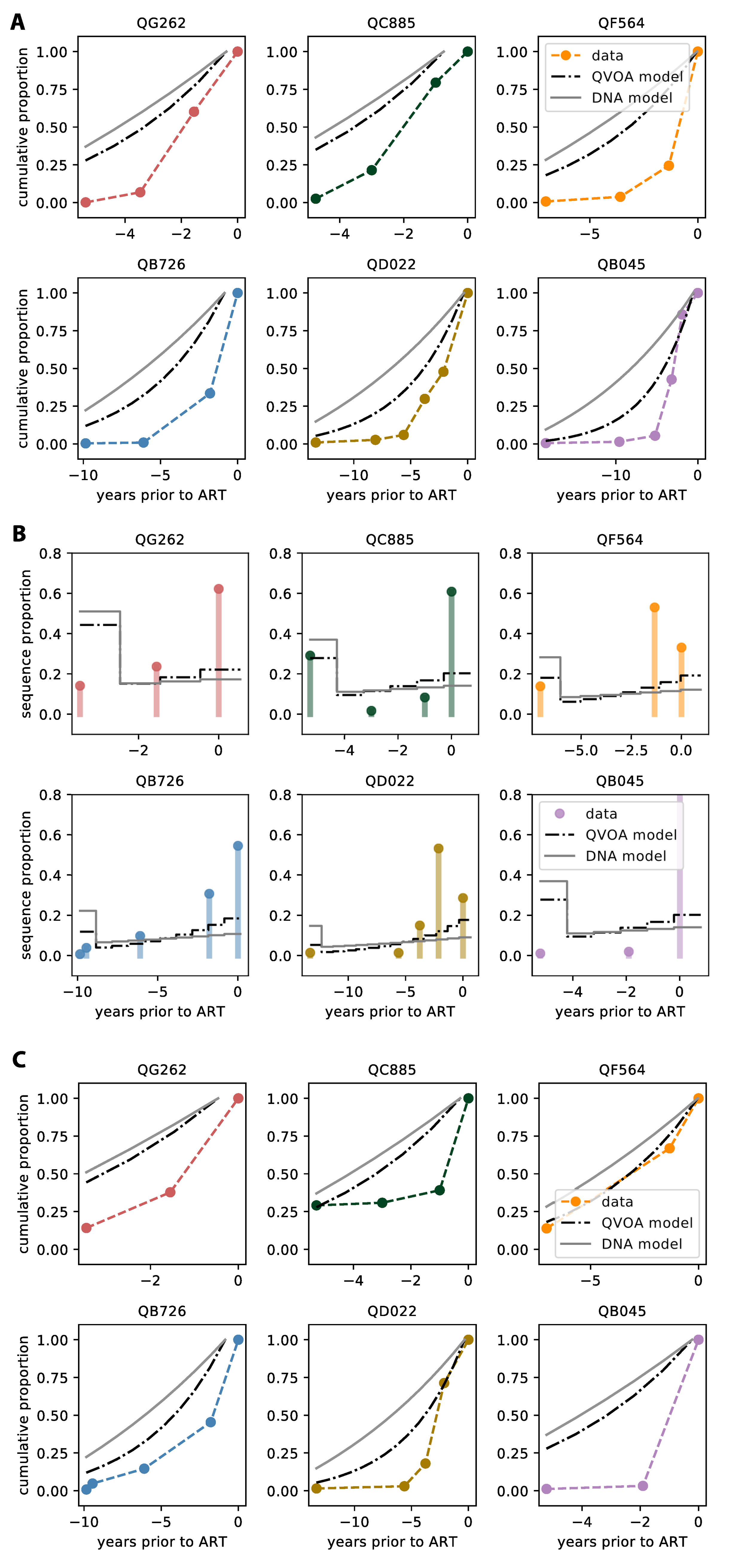

Supplement: S6 Fig — (A) Observed cumulative proportion of HIV gag sequences that contribute to the HIV DNA reservoir over time prior to ART (circles connected by dashed lines colored according to individual subject ID). Proportions of HIV gag sequences that contribute to the reservoir over time as predicted by the cumulative models using on-ART estimated decay rates of 44-month half-life based on QVOA measurements (black dashed line) and the 140-month half-life based on total HIV DNA measurements (grey solid line). Comparison of the models to the observed data was performed using a non-parametric Kolmogorov-Smirnov test and the difference between the models and the observed data was significant (p<0.01) for all individuals using either half-life model, confirming that the constant seeding and continuous decay model is unlikely to explain the observed data. (B) Comparisons of cumulative models to the experimental proportions of env sequences estimated to contribute to the reservoir from each year of infection in 6 individuals. Models based on a half-life of 44 months (dashed black line) or 140 months (grey line) differ with length of infection. Experimental data suggests the reservoir is comprised of a larger number of sequences created near the time of ART initiation than would be expected from models using either clearance half-life. (C) Observed cumulative proportion of env sequences that contribute to the HIV DNA reservoir over time prior to ART are compared to proportions of env sequences predicted by the cumulative models (graph details as in panel A). (TIF) [file ppat.1008286.s006.tif]

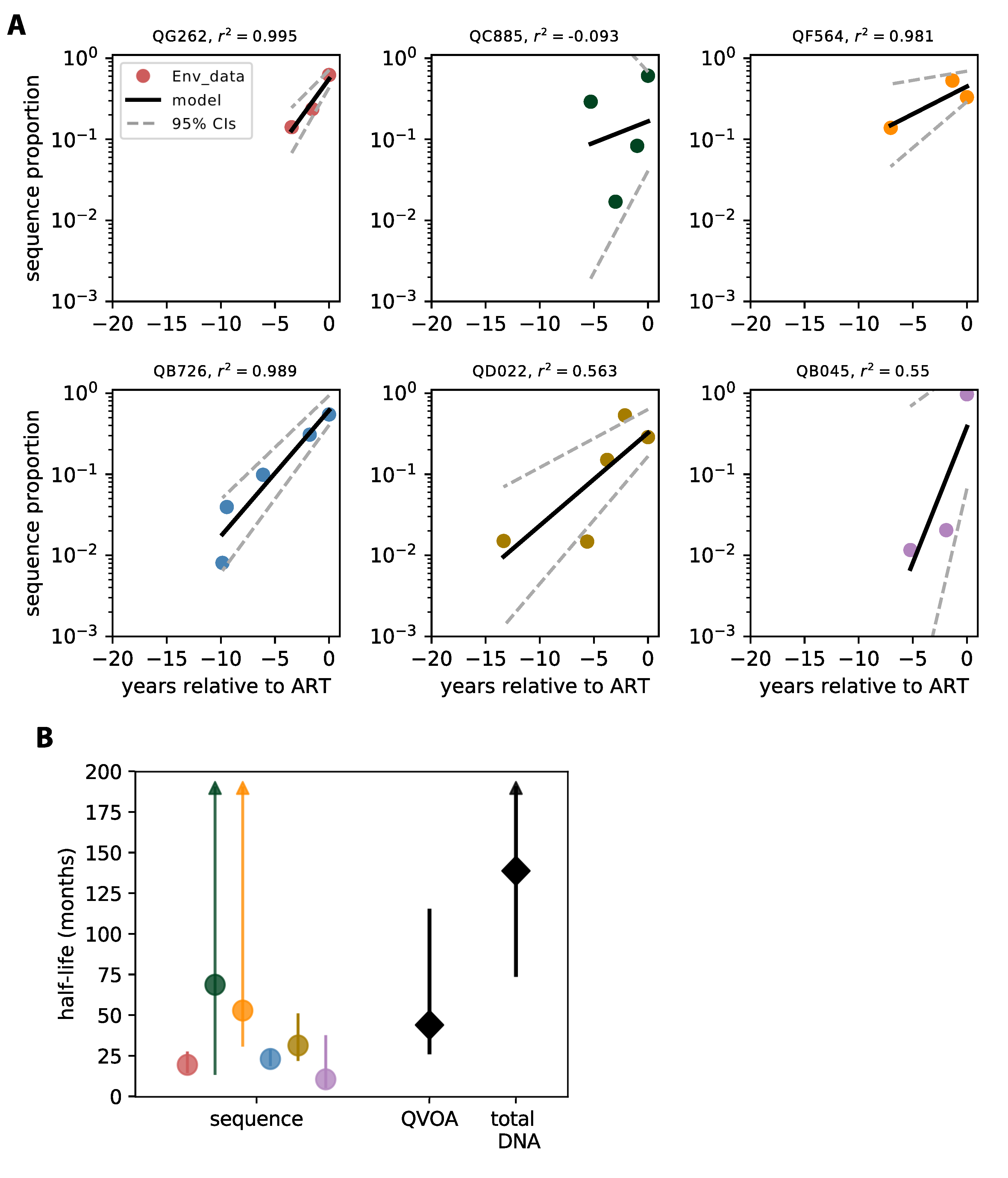

Supplement: S7 Fig — (A) Log-linear regression plots of relative sequence abundance show clearance of env sequences over time during untreated infection in each of the 6 subjects. (B) Observed total HIV env DNA pre-ART sequence half-lives for each individual based on the log-linear regressions are shown: circles (color by subject ID as in A) indicate the median estimated half-life and lines show 95% confidence intervals (CIs) associated with each estimate. These are compared to previously reported on-ART reservoir population size half-lives based on replication-competent reservoir decay measured by QVOA [8] and total HIV DNA decay [10]: black diamonds indicate the median estimated half-life and lines show 95% CIs associated with each estimate. Arrows indicate a CI that is inclusive of an infinite half-life (no decay). (TIF) [file ppat.1008286.s007.tif]
